# Metabolomics as a Promising Resource Identifying Potential Biomarkers for Inflammatory Bowel Disease

**DOI:** 10.3390/jcm10040622

**Published:** 2021-02-06

**Authors:** Cristina Bauset, Laura Gisbert-Ferrándiz, Jesús Cosín-Roger

**Affiliations:** 1 Department of Pharmacology and CIBER, Faculty of Medicine, University of Valencia, 46010 Valencia, Spain; baupas@alumni.uv.es (C.B.); laura.gisbert@uv.es (L.G.-F.); 2 Hospital Dr. Peset, Fundación para la Investigación Sanitaria y Biomédica de la Comunitat Valenciana, FISABIO, 46017 Valencia, Spain

**Keywords:** inflammatory bowel disease, metabolomics, biomarkers

## Abstract

Inflammatory bowel disease (IBD) is a relapsing chronic disorder of the gastrointestinal tract characterized by disruption of epithelial barrier function and excessive immune response to gut microbiota. The lack of biomarkers providing early diagnosis or defining the status of the pathology difficulties an accurate assessment of the disease. Given the different metabolomic profiles observed in IBD patients, metabolomics may reveal prime candidates to be studied, which may help in understanding the pathology and identifying novel therapeutic targets. In this review, we summarize the most current advances describing the promising metabolites such as lipids or amino acids found through untargeted metabolomics from serum, faecal, urine and biopsy samples.

## 1. Introduction

Inflammatory bowel disease (IBD) is a chronic inflammatory alteration of the gastrointestinal tract which comprises ulcerative colitis (UC) and Crohn’s disease (CD). Although both pathologies are characterized by the presence of relapsing and remitting episodes associated with intestinal inflammation and epithelial injury, these diseases present notable differences. On the one hand, UC manifests as a diffuse inflammation affecting the mucosal layer and is mainly located at the rectum and colon. The most frequent symptoms of UC patients are rectal bleeding, weight loss, bloody diarrhoea, fever and, to a lesser extent, abdominal pain. On the other hand, CD is characterized by a localized and well-defined transmural inflammation affecting several layers, which can appear in any part of the gastrointestinal tract, although it affects more frequently the terminal ileum and colon. In this case, symptoms of CD patients present several differences depending on the disease location, behaviour and extent, the most frequent ones being abdominal pain, weight loss, diarrhoea, fatigue and fever [1].

The incidence and prevalence of IBD is continuously increasing each year, and it has become a crucial disease in both Western and newly industrialized countries that needs to be treated as an important public health pathology [2]. Despite all the great progress that has been achieved during the last two decades, the aetiology of IBD is still not well known. To date, it is assumed that IBD is a multifactorial disease in which different factors such as genetic, environmental, microbial and immunological are responsible for the onset of this disease [3]. Therefore, it is still challenging to better elucidate and define the aetiopathogenic mechanisms involved in IBD in order to stablish new pharmacological targets with the aim to obtain a pharmacological treatment that induces a complete remission of the pathology.

Once an IBD patient is diagnosed, the specific subclassification of IBD is sometimes complicated, since it is still difficult to differentiate between CD, UC and other gastrointestinal diseases. In addition, the clinical symptoms of IBD patients do not often correlate with the disease activity, which makes even more difficult to stablish a precise diagnosis and a pharmacological treatment for each patient [4]. Therefore, clinicians need more tools in order to stablish a “gold standard” test to diagnose, assess the disease severity or even evaluate the response to the pharmacological treatment. With this goal in mind, during recent decades, the search of IBD biomarkers has been extensive, and several of them have been introduced into the clinical practice. Among these biomarkers, we can find some serological antibodies such as perinuclear anti-neutrophil cytoplasmic antibodies (pANCA), anti-saccharomyces cerevisiae antibodies (ASCA), antibodies against exocrine pancreas (PABs), circulating non-coding RNAs including mi-RNAs and lncRNA, C-reactive protein (CRP), cathelicidin, Trefoil factor 3 (TFF3) [5]. Nevertheless, the use of most of the reported biomarkers is still controversial, and unfortunately, there is still a lack of a set of biomarkers that allows clinicians to better diagnose or evaluate the clinical disease activity in IBD patients.

Among all the omics that have been recently developed, metabolomics is one of the most recent technique that has bounced into IBD. This is due to the fact that metabolomics has the potential not only to identify specific biomarkers for diagnosis and monitoring the disease behaviour, but also to elucidate specific molecular mechanisms involved in this pathology. In fact, this omic is being used in the study of several diseases such as cancer, diabetes, multiple sclerosis, cardiovascular ischemia. Metabolomics is the large-scale study of small molecules, known as metabolites, in biological samples, which, in case of IBD patients, can be biopsies, stool, plasma and urine. Although most of the studies performed so far have used the proton nuclear magnetic resonance (^1^H-NMR), advances in methodology and mass spectroscopy (MS) have also introduced this technique in the analysis of the metabolomic profiles in biological samples [6].

Despite all the metabolomic studies performed in IBD patients so far, the specific metabolic alterations in these patients still remain unclear. Nevertheless, it is clear that the identification of specific metabolic signatures might be extremely useful in the diagnosis of both UC and CD and also in the evaluation of the disease activity. Therefore, in the present review, we describe all the different metabolites that have been differentially detected in both UC and CD patients, and we try to clarify whether these compounds might represent promising biomarkers for IBD patients or even the components of metabolomic pathways, which can be considered as pharmacological targets for IBD treatment.

## 2. Tricarboxylic Acid Cycle (TCA) Intermediates

The tricarboxylic acid cycle (TCA) cycle, also named as the Krebs cycle, constitutes a key protagonist in cell metabolism. It includes a loop of consecutive reactions that obtain reducing equivalents NADH and FADH2, which are essential to transfer electrons to the mitochondrial respiratory chain, also named as the electron transport chain (ETC). This metabolic pathway involves different specific metabolites such as citrate, isocitrate, α-ketoglutarate (α-KG), succinyl-CoA, succinate, fumarate, malate and oxaloacetate [7]. In this section, we describe all the metabolic alterations of these TCA cycle intermediates detected in IBD patients. In Table 1, we have summarized all these reported metabolic changes including the alteration observed, the group of patients analysed, the type of sample and technique used in each study.

Due to the fact that serum and urine samples can be easily obtained, most metabolomic studies were performed using these biological samples. Growing evidence point to an impaired TCA cycle in IBD patients. In fact, Schicho and colleagues analysed the metabolome of serum, plasma and urine samples of UC, CD and healthy controls, and they reported lower levels of citrate in both CD and UC patients compared with control in serum and urine samples, while no differential levels in citrate were detected in plasma samples. In addition, these authors also demonstrated that citrate levels in serum can differentiate between CD and UC, since CD patients showed higher levels of this metabolite [8]. In line with this, Stephens and colleagues analysed the metabolome of urine samples of CD, UC and healthy controls, and they showed a significant reduction in the levels of the TCA cycle intermediates citrate, succinate and aconitate in IBD patients compared with healthy controls [9]. More recently, Scoville and colleagues analysed the metabolome of the serum of non-IBD, CD and UC patients and also reported a significant decrease in all the TCA cycle intermediates analysed in CD patients compared with non-IBD or UC patients. Specifically, they showed reduced levels of citrate, aconitate, α-ketoglutarate, succinate, fumarate and malate [10].

This differential abundance of TCA cycle intermediates would allow us to (a) discriminate between non-IBD and IBD patients, (b) discriminate between IBD patients and other inflammatory diseases and (c) evaluate the disease activity. Indeed, Alonso and colleagues analysed the metabolome of the urine of several patients with immune-mediated inflammatory diseases (IMIDs), including IBD, and controls. In that study, authors reported reduced levels of citrate in both CD and UC patients compared with healthy controls. In the same study, authors go even further and point to citrate as a possible biomarker of the disease activity specifically in CD patients, since the concentration of this metabolite was significantly lower in those CD patients with a higher degree of disease activity [11]. In this line, Dawiskiba and colleagues collected both serum and urine samples of both active and in remission IBD patients and control subjects and analysed the metabolomic profiles in those samples. In serum samples, they reported a significant decrease in levels of citrate in IBD patients compared with control and increased levels of this metabolite in active IBD patients compared with IBD patients in remission. In addition, they reproduced these observations with the urine samples, and they also detected reduced levels of succinate in IBD patients versus control subjects. However, in this case, they reported lower urine levels of succinate in active IBD patients compared with IBD patients in remission [12]. These studies point to these metabolites as possible biomarkers for monitoring the clinical behaviour of the pathology in IBD patients.

Besides serum and urine samples, faecal samples have also been used in order to analyse the metabolome in IBD patients. In this case, unlike serum and urine samples, there are no significant differences in TCA cycle intermediates measured in faecal samples. In fact, although Marchesi and colleagues showed notable differences of a typical ^1^H-NMR spectra of faecal extracts obtained from healthy volunteers and patients with CD and UC, the authors revealed no significant differences between the groups analysed regarding levels of succinate [13]. In line with this, Martin and colleagues also reported no changes in the levels of succinate nor citrate in faecal samples of paediatric CD and UC compared with healthy controls [14]. These differences observed between the metabolomic profiles in faecal samples and those observed in the serum and urine samples are probably due to the involvement of the microbiota, one of the central protagonists in IBD pathology.

Unfortunately, there are few studies that have analysed the metabolomic profiles specifically in intestinal tissues of these patients. Balasubramanian and colleagues obtained intestinal biopsies from both UC and CD patients, and they revealed a significant decrease in levels of succinate in both UC and CD patients compared with healthy controls. However, although they divided the IBD patients in active and remission, they did not observe significant differences between them [15]. Of interest, Ooi and colleagues compared the metabolome of the damaged and normal tissue of intestinal biopsies from UC patients, and they showed that levels of succinate, citrate, fumarate, malate and isocitrate were significantly lower in the damaged area compared with the normal tissue [16].

To our knowledge, there are only two studies that have used intestinal surgical resections from CD patients in order to analyse metabolomic alterations. In the first study, the authors reported a significant increase in succinate in CD patients compared with control subjects [17]. In addition, in a second study, the same authors expanded their observations, and they have recently reported that succinate levels are even increased in CD patients with a penetrating behaviour (B3-CD patients) compared with CD patients with a stricturing behaviour (B2-CD patients) [18]. This difference in one TCA cycle intermediate reveals the importance of studying the metabolomic patterns specifically in intestinal resections of IBD patients.

Due to the emerging development of several omics, the most recent studies try to integrate the results obtained by different omics in order to have a more complex observation of IBD patients. In this sense, epigenetics can also modify the cell metabolism, which in turn, modulates the metabolite levels in the tissue. In fact, the histone succinylation, lactylation and crotonylation rely on substrates succinyl-coA, lactate and crotonyl-coA levels, respectively. In addition, another TCA component, such as α-ketoglutarate, can serve as substrate, donor, cofactor or competitive inhibitor for various epigenetic enzymes [19]. On the other hand, given the great importance of the intestinal microbiota in IBD patients, most of these current studies try to correlate the metabolomics changes with all the alterations observed in intestinal microbiota. Of interest, it has been recently reported that succinate levels are increased in stool samples of paediatric CD patients infected with *Clostridioides difficile* compared with paediatric CD patients without infection. In this elegant study, the authors associate for the first time a metabolomic alteration with a specific bacterium [20]. In line with this, it has also been recently reported that succinate levels of intestinal aspirates from paediatric IBD patients positively correlates with the invasion of *Escherichia Coli* [21]. Nevertheless, there is still a gap between all the findings related to the intestinal microbiota and all the different metabolomics profiles reported so far. Therefore, further studies are also needed in order to integrate all the big data generated by these omic approaches, including the microbiota information, and to elucidate the specific molecular mechanisms involved in this pathology.

## 3. Lipids

The term lipid includes a large amount of molecules with a wide variety of different structures and biological functions affecting cell membranes, metabolic processes and signalling pathways, and acting as energy storage sources [22]. Lipids are classified in eight different categories: fatty acids, sterol lipids, sphingolipids, sphingolipids, glycerolipids, glycerophospholipids, prenol lipids, saccharolipids and polyketides [22]. In the following section, we discuss the alterations in lipid profiles detected in IBD patients following the above classification.

### 3.1. Fatty Acids

Firstly, fatty acids constitute a group of compounds that play important roles in the regulation of physiologic and metabolic pathways. Their specific role in inflammation seems to be dual, since some of them exhibit proinflammatory functions, while others possess anti-inflammatory properties. Alterations in their physiological levels are traduced in modifications in lipid cascades and important metabolic signalling pathways that modulate the inflammatory response [23]. All the reported metabolic alterations described in IBD patients regarding fatty acids are summarized in Table 2.

Long-chain (LCFAs), medium-chain (MCFAs) and polyunsaturated fatty acids (PUFAs) have been found altered in serum, plasma, urine, faecal and colonic mucosa samples of IBD subjects compared to non-IBD controls. Lai and colleagues reported decreased levels of LCFAs such as docosahexaenoic acid, linolenic acid and arachidonic acid and MCFAs such as pelargonic acid and caprylic acid in serum of CD patients [23]. In line with this, Daniluk and colleagues also found in serum of paediatric IBD patients decreased levels of the LCFAs acids arachidonic acid and docosahexaenoic acid [24]. Moreover, Scoville and colleagues reproduced those observations and authors described reduced levels in serum samples of several metabolites related to LCFAs, branched chain and monohydroxy fatty acids metabolism in the IBD group in comparison to healthy controls. The same group also demonstrated differences in LCFAs-related pathways in CD patients when compared to UC or control subjects [10]. In addition, authors also highlighted a previous study that evidenced that lipid profiles of patients receiving effective treatment, such as infliximab, were similar to controls over time [25].

On the other hand, Jansson and colleagues analysed the metabolome using faecal samples. Of interest, in their study, they distinguished between predominantly ileal CD (ICD) and predominantly colonic CD (CCD) patients, and they found increased amounts of fatty acids such as arachidonic acid, linoleic acid, oleic acid, stearic acid, palmitic acid and 6Z-, 9Z- and 12Z-octadecatrienoic acid in ICD vs. CCD and control individuals [26]. This apparent paradox between the increased levels found in faecal samples and decreased levels in serum samples might be due to deficiencies in gut absorption, which commonly occurs as a result of mucosal inflammation. Nevertheless, Weng and colleagues also performed an untargeted metabolomic analysis of faecal samples, and they found that LCFAs such as arachidic acid, oleic acid and tridecanoic acid, as well as MCFAs such as sebacic acid and isocaproic acid, were decreased in UC and CD patients compared with controls [27].

Other fatty acids that have also been detected in serum of IBD patients are acylcarnitines. However, in this case, it is important to mention that the already published information is still controversial. On the one hand, decreased levels of these fatty acids were observed in the serum of CD patients compared with UC and healthy controls [10]. On the other hand, Lai and colleagues found, also in serum samples, increased levels of certain acylcarnitines such as propionylcarnitine, butyrylcarnitine, isovalerylcarnitine and heptanoylcarnitine [23]. Hence, to address this controversy, future studies are required in order to determine the real levels of these fatty acids and their role in IBD pathogenesis.

Up to this point, most of the evidence points to a reduction in LCFAs and MCFAs in IBD patients compared with non-IBD patients [10,23,24]. This fact, together with increased levels of acylcarnitine derivatives, suggest an enhanced β-oxidation of fatty acids in IBD patients, since the rate-limiting step in the β-oxidation of fatty acids is the availability of acylcarnitines [23]. Indeed, increased β-oxidation in IBD patients may be due to higher energy demand in order to recruit immune cells to face inflammation, which results in higher LCFAs consumption. As a consequence to this higher energetic demand and the reduced levels of these fatty acids, the immune response might be aggravated [10,28]. For instance, NF-κB activation involves the production of proinflammatory cytokines [29]. This activation is inhibited when MCFAs and LCFAs bind and activate PPAR-γ and PPAR-α receptors, triggering an anti-inflammatory effect [30].

The metabolomic analysis performed by Lai and colleagues and Daniluk and colleagues showed reduced levels of arachidonic acid in serum of adult CD and paediatric UC patients, respectively [23,24]. In addition, Jansson and colleagues analysed the arachidonic acid levels in faecal samples of ICD patients, and they found increased levels of this metabolite in ICD patients, which may be due to malabsorption [26]. At this point, it is important to consider that the inflammatory process implies an increased consumption of fatty acids and increased concentrations of arachidonic acid and docosahexaenoic acid, due to a higher expression of phospholipase A2, which hydrolyses phospholipids into arachidonic acid. Indeed, both arachidonic acid and docosahexaenoic acid are also responsible of increasing the biosynthesis of PUFAs, which in turn, regulate the inflammatory response and play an anti-inflammatory role [24]. On the other hand, the key role of arachidonic acid in inflammation has also been demonstrated, since the expression of the intracellular adhesion molecule ICAM-1 is increased by arachidonic acid, favouring the inflammatory response by recruiting leukocytes [26,31]. Furthermore, arachidonic acid, together with linoleic acid, increase the production of prostaglandins, mediators in the immune response. Nevertheless, in the analysis performed by Jansson and colleagues, prostaglandins were increased in the control cohort compared with IBD patients. Authors stated that those reduced levels of prostaglandins in the CD cohort may be due to malabsorption of both arachidonic and linoleic acids [26]. Even though prostaglandin levels are lower in CD patients when comparing patients in remission with active CD–patients, higher levels of prostaglandins are found in the last ones, especially the prostaglandin PGE2. This prostaglandin is responsible of activating Th17 lymphocytes, which characterize CD pathology, by activation of dendritic cells and further production of IL-23 by them [32]. Nevertheless, there are not yet studies that demonstrate the correlation between increased levels of prostaglandins with an increase in linoleic and arachidonic acids.

Another type of fatty acids commonly disturbed in IBD patients are short-chain fatty acids (SCFAs). These fatty acids are products of the fermentation of complex carbohydrates by anaerobic gut bacteria [13,33]. According to the number of carbons in their structure, different types of SCFAs are described being the most abundant the acetate, propionate and butyrate with two, three or four carbon atoms, respectively [34]. Several studies conclude that SCFAs produced by gut microbiota are preferentially used as energy source by colonic epithelial cells to promote cell growth and modulate the immune response in the gastrointestinal tract by enhancing epithelial barrier [35,36], although the mechanism is not well established yet [37]. This energy is also used to absorb sodium in the colon [38]. Previous studies suggest that the impaired metabolism of SCFAs in IBD patients might be due to a defective uptake from faecal effluents caused by a disruption of the sodium–potassium pump related to alterations in mucosal cell membranes [15]. SCFAs have been found reduced in faecal and urine extracts of IBD patients in many studies [9,13,33,39]. In fact, lower levels of the SCFAs butyrate and propionate were found in faecal samples of IBD patients compared with healthy controls [13,39]. In the study performed by Marchesi and colleagues, authors could distinguish between UC cohort and CD cohort, since UC patients did not show such decreases compared with CD patients. Thus, CD group of patients seemed to be more affected by inflammation than UC patients [13]. In addition, Stephens and colleagues also found, in this case in urine samples, reduced levels of acetate in IBD patients compared with healthy subjects [9]. Thus, decreased levels of SCFAs can be associated with the disruption of fermentative gut microbiota community due to gut mucosal inflammation in IBD patients [39]. Besides SCFAs, certain MCFAs such as caprylic acid are also produced by gut microbiota, as described in the study performed by Franzosa and colleagues, where they found in faecal samples reduced levels of caprylic acid [33].

Finally, the last type of fatty acids found altered in IBD patients are PUFAs. In this aspect, Scoville and colleagues found in serum samples decreased PUFAs levels in IBD subjects when compared to non-IBD controls. These levels were even lower in CD patients compared with UC patients [10]. In line with this, Franzosa and colleagues found in faecal samples increased levels of eicosatrienoic, an omega-3 fatty acid, and docosapentaenoic, an omega-6 fatty acid, which are considered as anti-inflammatory compounds [33]. As mentioned above, these increased levels in faeces may indicate a lack of absorption in the gut due to inflammation. It is important to take into account that, as previously stated in this section, PUFAs play many roles related with the intestinal inflammatory response, since they are responsible of synthetizing inflammatory mediators, such as eicosanoids, and they regulate cell membranes of immune cells [10,40]. In addition, they possess a bactericidal activity, since they are capable of affecting bacterial cell membranes [41]. Taking everything together, it is clear that levels of fatty acids are commonly altered in IBD contributing to the inflammatory state, which characterizes this pathology. Hence, fatty acids constitute an important group of metabolites, which can be considered promising biomarkers but further studies including larger and wider number of patients are still needed.

### 3.2. Sterol Lipids

Sterol lipids, derived from condensation of isoprene subunits, constitute a huge class of lipids [22]. In this review, among all the types of sterol lipids described, we specifically focus on bile acids and cholesterols due to their impact on IBD. Alterations of those sterol lipids described in IBD patients are synthetized in Table 3.

Regarding the bile acids pathway, the role of these sterol lipids is to facilitate lipids digestion, and afterwards, they are transformed by commensal microbiota to secondary bile acids [33]. In the metabolomic analysis performed by Franzosa and colleagues using faecal samples, secondary bile acids, such as lithocholate and deoxycholate were found in lower levels in CD patients in comparison to healthy controls, while in contrast, primary bile acids, such as cholate and chenodeoxycholate, were increased in CD patients compared with healthy controls. These differential levels observed in IBD patients can be explained, since primary bile acids are accumulated in the gut due to the fact that the presence of microorganisms responsible of undergoing such deconjugations is reduced and/or their function is impaired in the intestine of IBD patients [33]. In line with this, Weng and colleagues also showed in faecal samples of IBD patients lower levels of bile acids, such as lithocholic acid, chonodeoxycholate and taurolithocholic acid, in comparison to controls. Specifically, glycochenodeoxycholate and glycolithocholic acid were decreased in UC patients [27]. At this point, it is important to consider that vitamin D receptor is activated by lithocholic acid, which protects from bile acid toxicity by promoting synthesis of proinflammatory cytokines and dendritic cells differentiation [43,44]. Moreover, bile acids can suppress immune response when they activate the G protein-coupled receptor 5, because this receptor inhibits cytokines production [45,46]. Therefore, when bile acids are decreased, their anti-inflammatory role is not performed, and the immune response is stronger.

Reinforcing those observations, Scoville and colleagues have recently analysed bile acids, and they also found increased levels of these metabolites in serum of CD patients. In fact, primary and secondary bile acids were increased in CD patients when compared to controls. In contrast, sulphated bile acids such as glycolithocholate sulphate or taurocholenate sulphate were reduced in CD patients in comparison to healthy controls. Interestingly, UC patients exhibited reduced levels of primary bile acids glycochenodeoxycholate glucuronide and secondary bile acids [10]. This difference between both diseases might be due to the fact that CD is characterized by small bowel malabsorption, while in UC, the small bowel is not often affected. Although malabsorption has also been argued, the fact that authors found alterations in both CD and UC patients suggest that malabsorption in the small bowel is not the only cause of such alterations and that bile acid pathways have different implications in IBD [10]. Indeed, some studies suggest that the increased levels of bile acids in serum of CD subjects are due to an increased bile acid production in order to relieve their lack given the malabsorption problems [25].

Of interest, Jansson and colleagues found higher levels of glycocholate in faecal samples of CD subjects in comparison with healthy controls. In addition, they reported higher levels of certain bile acids specifically in ileal-CD patients compared with colonic-CD patients, such as taurocholate, 3α, 7α, 12α-trihydroxy-5β-cholanate and chenodeoxyglycocholate. It is important to consider that the removal of distal ileum by surgery triggers an accumulation of bile acids given the fact that they are mainly absorbed in that part of the intestine [26]. In fact, some studies have reported alterations in bile acids composition in patients with ileal resection [47]. Hence, some of the metabolites altered in IBD, especially in CD, are not caused by the pathogenic process itself, but they result from the associated small bowel resection [10].

On the other hand, cholesterols are another type of sterol lipids that have also been differentially found in IBD patients. Franzosa and colleagues reported lower levels of cholesterol in the CD group in contrast with healthy individuals in faecal samples [33]. A deeper analysis of the specific cholesterol molecules performed by Williams and colleagues revealed that low-density lipoprotein (LDL) cholesterol and high-density lipoprotein (HDL) cholesterol were reduced in serum samples of IBD patients in comparison to the controls subjects. Furthermore, in the same study, authors also compared between CD and UC patients, and both LDL and HDL cholesterols were even more decreased in CD patients when compared to the UC group. In contrast to LDL and HDL levels, very low-density lipoprotein (VLDL) cholesterol was increased in IBD patients compared to controls, as well as in CD versus UC patients. Most of the studies performed so far conclude that IBD is characterized by several lipid metabolism alterations, which are strictly related to chronic inflammation [42]. In fact, the pro-inflammatory cytokines, such as TNF-α, IL-1 and IFN-γ, which are abundant in IBD patients, are responsible of inhibiting lipoprotein lipase (LPL) expression [48,49]. This LPL protein hydrolyses circulating triglycerides into chylomicrons and VLDL into free fatty acids and glycerol, which are further converted in HDL and LDL [42]. In addition, the lack of HDL also aggravates the inflammatory state, since its main protein, apolipoprotein A-I, binds to T-lymphocytes and prevents monocyte activation, inhibiting TNF-α production [50,51]. This anti-inflammatory effect of HDL is strongly reinforced by accumulative evidence that reveals a disturbance in the composition of lipoproteins in IBD patients [52,53]. Therefore, given the key role of the cholesterol in inflammation, the specific analysis of these sterol lipids might provide important information about specific molecular mechanisms involved in the aetiopathogenesis of IBD.

### 3.3. Sphingolipids, Glycerophoshpolipids, Glycerolipids and Prenol Lipids

Sphingolipids, as lipidic compounds, act as signalling molecules and are part of cell membranes composition [54]. Structurally, they are constituted by a sphingoid base, such as sphingosine, which is linked, through an amide bond, to an LCFA to form a ceramide. Several studies have documented alterations in ceramides and other derivatives such as lactosylceramide, sphingomyelin or sphingosine 1-phosphate in IBD patients. In this section of the review, alterations in the metabolism of sphingolipids, glycerophospholipids, glycerolipids and prenol lipids detected in IBD patients are summarized in Table 4.

In the metabolomic analysis performed by Franzosa and colleagues, sphingolipids, such as ceramide and sphingomyelin, were found increased in the IBD cohort when compared to healthy subjects in faecal samples [33], whereas Scoville and colleagues reported reduced levels of sphingomyelin in serum of IBD patients [10]. It is important to mention that growing evidence indicate that the inflammatory process might be, in part, promoted by the accumulation of sphingolipids due to an alteration of the sphingolipid metabolic pathway in IBD pathogenesis [55,56]. Indeed, sphingomyelin and phosphorylcholine are lipidic cell membrane components, which are synthetized from choline [57], an essential nutrient [58], whose deficiency has been associated with some diseases such as non-alcoholic fatty liver [59]. In this line, numerous studies reported lower levels of this metabolite in serum samples of IBD patients [8,12,42]. In addition, Williams and colleagues found differences between CD and UC patients showing reduced levels specifically in CD subjects [42]. In line with this, Balasubramanian and colleagues reinforced these observations, since they found reduced levels of choline in colonic mucosa of IBD patients compared to controls. Moreover, authors also reported that active CD patients showed even reduced levels of choline compared with patients in remission [15]. On the other hand, it has also been reported that choline acts as a scavenge for free radicals, thus, a lack of choline aggravates inflammation in IBD [42,60]. Furthermore, some studies demonstrate that lymphocyte genes are overexpressed in individuals with reduced levels of choline, promoting the inflammatory response [61]. In addition, choline is also present in the structure of platelet activating factor (PAF), an inflammatory mediator [62]. Due to the inflammatory response that characterizes IBD, these patients show an increase in PAF, which in turn, implies an increased demand of choline for its structure [63]. In fact, transmural inflammation, which characterizes CD patients, might imply higher demand of choline in comparison with UC patients [42]. Hence, this increased requirement of choline might be associated to the decreased levels of this metabolite found in CD patients.

The alterations in sphingolipids have also been reported in paediatric IBD patients [64]. Indeed, Daniluk and colleagues performed a metabolomic analysis using serum of a paediatric cohort suffering IBD, which were recently diagnosed and not treated yet. In that study, authors found decreased levels of glycerophospholipids, such as lysophosphatydilcholine, phosphatydilcoline, phosphatydilethanolamine and lysophosphatidylethanolamine, in paediatric IBD patients and reduced levels of certain sphingolipids, such as sphingomyelin, specifically in CD patients [24]. Of interest, Daniluk and colleagues reported for the first time increased levels of the sphingolipid lactosylceramide in CD patients compared to the UC cohort, becoming a promising biomarker, which might help in the specific diagnosis of CD or UC. In line with this, a recent study performed by Filimoniuk and colleagues also found in serum of non-treated IBD children increased levels of lactosylceramide in CD compared with UC subjects [65]. Authors suggested that the increased levels of lactosylceramide in the CD cohort may be due to two different possibilities: these patients suffer alterations in the sphingolipid metabolism, or there is an unknown source of this metabolite, which is abnormally increasing its concentration. At this point, it is important to consider that lactosylceramide is a metabolite highly involved in the inflammatory response, and it is found in many inflammatory cell types such as granulocytes, monocytes or platelets. Moreover, it participates in cell–cell interactions, generation of nitric oxide, phagocytosis or as an intracellular mediator [24]. This metabolite, through generating superoxide species, stimulates the expression of ICAM-1 on endothelial cells surface, which favours the adhesion of neutrophils [66,67]. In fact, neutrophils show high levels of lactosylceramide and the proinflammatory cytokine TNF-α favours, in turn, the lactosylceramide production [68]. In addition, lactosylceramide is also responsible of stimulating phospholipase A2 and arachidonic acid release [69], a lipid mediator in inflammatory disease as previously explained. Therefore, it is clear that lactosylceramide might represent a promising biomarker for IBD patients, which needs to be validated in bigger cohorts of IBD patients and better studied in order to characterize its specific role in IBD.

Lai and colleagues found that CD patients exhibit decreased levels of sphingosine 1-phosphate (d16:1) in contrast to healthy controls [23]. This metabolite participates in inflammatory responses, as well as regulatory activities in several biological processes [70]. A study performed by Suh and colleagues, in which they determined sphingolipids profiles and the gene expression related to sphingosine 1-phosphate in an IBD cohort, demonstrated increased transcriptomic signatures in patients with active IBD in comparison to normal transcriptomic levels in IBD patients with remission. In addition, increased amounts of pro-apoptotic and pro-inflammatory sphingolipids were also found in this study [71]. Regarding the levels of these metabolites specifically in the intestinal tissue, Balasubramanian and colleagues found decreased levels of glycerophosphorylcholine, a glycerophospholipid that is part of the cell membrane, in colonic mucosa of IBD patients in comparison to the control group. This study reflects that inflammation affects mucosal cell membranes composition [15].

Regarding glycerolipids, the levels of glycerol have also been differentially detected in IBD patients. In fact, Scoville and colleagues found decreased levels of glycerol in serum samples of CD patients in comparison with healthy controls [10]. In addition, Marchesi and colleagues found increased glycerol levels in faecal samples of CD compared with UC patients and control subjects [13]. Finally, Franzosa and colleagues detected decreased levels of triacylglicerols and prenol lipids such as triterpenoids in faecal samples of IBD subjects when compared to its relative controls, contributing to the perturbations of lipid metabolism in the IBD pathology [33]. Nevertheless, given the scarce number of studies analysing these type of lipid metabolites, further studies are needed in order to better characterize their levels in these patients, since the lipid metabolism plays a key role in this pathology, and it is very probable that one of these lipid metabolites might become an interesting biomarker for IBD patients.

## 4. Amino Acids

Amino acids are defined as organic substances that contain both amino and acid groups. Although numerous amino acids have been described so far, only 20 serve as building blocks for protein synthesis. Those which constitute proteins are classified as protein amino acids, while those which do not constitute polypeptidic chains are classified as nonprotein amino acids [72]. In Table 5, we summarize all the studies that have demonstrated differential levels of amino acids in IBD patients specifying the type of sample and the technique used in each study.

Firstly, it is important to take into account that due to inflammation, the epithelial barrier protection is disrupted leading to the malabsorption of nutrients in the gut and resulting in increased levels of amino acids in faeces of IBD patients [13,39]. Indeed, numerous studies using faecal samples confirmed this premise, since they reported increased levels of amino acid in IBD patients [13,26,39]. For instance, Bjerrum and colleagues reported increased levels of branched-chain amino acids such as isoleucine, leucine and valine, as well as other amino acids such as alanine, lysine, tyrosine in faecal samples of IBD patients compared to its relative controls [39]. On the other hand, as many studies conclude, the concentration of several amino acids in serum samples is usually decreased in these patients [10,23,42]. This difference depending on the type of the sample analysed demonstrates that patients suffering IBD exhibit an intensified catabolism of proteins as well as decreased absorption of amino acids in the gut due to inflammation and increased intestinal permeability [42]. Furthermore, it is important to consider that these patients show a decrease in the gut microbiome taxonomic diversity in comparison to healthy controls [33,73]. Most studies have reported changes in microbiota of IBD patients correspond with altered pathways and metabolites due to the fact that gut microorganisms are responsible of transforming dietary products into essential compounds for our metabolism [33,74,75,76]. Indeed, amino acids and their derivatives are the most common affected metabolites in this context. These compounds usually constitute ligands of transcription factors or certain receptors playing an important role in the inflammatory process and regulating the immune response [23].

Tryptophan is an essential amino acid whose levels are disturbed in IBD patients. In fact, Jansson and colleagues found increased levels of this amino acid in faecal samples of ICD subjects compared with controls [26]. In line with this, Schicho and colleagues used urine samples, and they also found increased levels of tryptophan in the UC cohort [8]. Of interest, Nikolaus and colleagues reported decreased levels of this amino acid in serum of IBD patients [77]. These results shed light to the importance of specifying the type of sample used to undergo the metabolomic analysis. In the case of urine and faecal samples, it is important to take into account that external factors such as dietary intake may influence in the variability of the obtained results, and they cannot exactly be correlated with the pathology [8].

Besides from tryptophan itself, more recent metabolomic analyses have revealed alterations in derivatives from tryptophan metabolism. Indeed, the study performed by Lai and colleagues showed disturbances in indolic derivatives, compounds that are produced from tryptophan by commensal microbiota. Specifically, they reported decreased levels of indole-3-propionic acid and indole-3-acrylic acid, while increased levels of 3-methylindole in CD patients [23]. In this sense, it is well known that commensal microbiota produce tryptophan decarboxylases that convert tryptophan into tryptamine and indole derivatives among others [78]. Such indole derivatives are known to bind aryl hydrocarbon receptor and stimulate IL-10 activity, an anti-inflammatory cytokine that inhibits the gene expression of the proinflammatory cytokine TNF-α [79,80]. In the case of indole-3-acrylic acid, this metabolite binds pregnane X receptor and stimulates the gene expression of mucin 2, a structural protein from the intestinal epithelial barrier [81]. Hence, the decreased levels of these indole derivatives might be associated with the chronic inflammation observed in these patients. In addition, decreased amounts of 5-hydroxy-L-tryptophan (5-HTP) have been reported in the serum of CD patients [23], which affects serotonin levels triggering negative effects on intestinal permeability [82,83].

Another amino acid disturbed in IBD patients is phenylalanine. Nevertheless, levels of this amino acid are still an open question that must be addressed. On the one hand, according to the study performed by Lai and colleagues, phenylalanine was decreased in serum of CD patients in comparison to controls. Moreover, Bjerrum and colleagues and Jansson and colleagues found increased levels of phenylalanine but, in this case, in faecal samples of IBD patients. These results might be explained due to the fact that phenylalanine has an anti-inflammatory role, since it inhibits TNF-α production and enhances immune responses [84]. On the other hand, Dawiskiba and colleagues found increased levels of phenylalanine in serum of active IBD patients when compared to controls and IBD remission patients. In this case, the authors state that immune response inhibits phenylalanine-4-hydroxylase activity, the enzyme responsible of hydrolysing phenylalanine into tyrosine [12]. Altogether, additional studies are needed in order to clarify the exact levels of this amino acid in IBD patients.

Histidine metabolism has also been found perturbed in the metabolomic analysis performed by Lai and colleagues. In fact, ergothioneine, a histidine derivative converted by microbiota or obtained from diet, was found in lower levels in serum samples of CD patients [23]. This metabolite is an antioxidant and neuroprotective agent, which defends cells from mitochondrial DNA damage and lipid peroxidation [85]. Reduced levels of ergothioneine might be associated with lack or impairment of its transporter, the organic cation/carnitine transporter 1 (OCTN1), which has also been associated to other inflammatory pathologies such as rheumatoid arthritis [86,87]. Hence, ergothioneine seems to be a potential biomarker to distinguish between CD and UC, since its transporter is only expressed in small intestine [23]. In addition, Stephens and colleagues also found decreased levels of histidine, as well as lysine and asparagine, in urine samples of IBD patients in comparison with control individuals [9], and Dawiskiba and colleagues also found lower levels of histidine in serum samples of IBD patients [12].

The study performed by Jansson and colleagues found alterations also in the tyrosine metabolic pathway. They found that the metabolite dopaquinone was increased in faecal samples of CD patients in comparison to healthy controls. Dopaquinone is an intermediate of melanin formation, which comes from the oxidation of levodopa (L-DOPA) and, in turn, tyrosine [26]. In addition, other studies have demonstrated increased levels of L-DOPA in patients suffering IBD, another intermediate of this metabolite [88]. Unfortunately, there is not enough evidence to affirm that tyrosine pathway is involved or not in the pathogenesis of CD. Nevertheless, similar alterations have also been reported by other studies, which described an increase in the transcription of tyrosine metabolism genes in mononuclear cells from patients suffering CD [89].

On the other hand, Schicho and colleagues performed a metabolomic analysis using both plasma and serum samples, and they reported increased levels of isoleucine in serum samples from IBD patients in comparison to healthy subjects [8]. When using plasma samples, they found increased levels of lysine while reduced levels of valine and tyrosine. In both serum and plasma, they found decreased levels of ornithine, as well as urea in CD patients, and increased levels of arginine and methionine. Given the fact that most of them are intermediates of the urea cycle, authors state that IBD-related pathologies affect the urea cycle [8]. Additionally, some other studies also determined that there is a relationship between UC and increased levels of arginine [90].

In the specific case of glycine, it has been reported that there are increased levels of this amino acid in faecal [39] and urine samples [12] of IBD patients compared with healthy controls. It is important to consider that this amino acid plays a key role in the regulation of the inflammation [12]. When it binds to glycine-gated chloride channels, it inhibits the activation of immune cells such as neutrophils and macrophages, thus playing a cytoprotective and anti-inflammatory role [91].

Different levels of amino acids have also been reported specifically in intestinal mucosa. Indeed, Balasubramanian and colleagues revealed lower levels of the amino acids isoleucine, leucine, valine, glutamine, glutamate and alanine in colonic mucosa of IBD patients compared with healthy subjects [15]. The conversion of glutamine to alanine provides to the intestinal mucosa about one third of the required energy to accomplish the metabolic demand [92]. In this conversion, an amide group is formed, which is essential to synthetize membrane components of glycoproteins and amino sugars such as hexosamines [93]. Glutamine is also strictly related with the immune response, because it avoids bacterial attachment to mucosal cells by keeping IgA levels [94]. It also regulates inflammation by modulating IL-8 and TNF-α levels [15].

Of interest, Scoville and colleagues reported for the first time a set of amino acids that might be promising biomarkers in order to distinguish between CD and UC patients. In this study, authors reported decreased levels of amino acids such as leucine, lysine, valine, arginine, glutamine and serine in the serum of CD subjects when compared to its relative controls. In addition, they achieved interesting results, since they found that leucine, valine, serine and glutamine were even more reduced in CD patients than in UC patients and that there were no differences in the amino acid levels between UC and controls [10]. Moreover, Dawiskiba and colleagues found significant differences in alanine serum concentration between CD and UC patients [12]. Therefore, the specific analysis of these amino acids could help the complicated diagnosis between the two most common IBD pathologies.

The utility of the amino acids as biomarkers has been even expanded to evaluate the clinical state of IBD patients. In this sense, levels of branched-chain amino acids (isoleucine, leucine and valine) were discriminative between patients with active CD in comparison with patients in remission [15]. This last observation points to these amino acids as promising biomarkers in order to evaluate the disease activity of CD patients. Taking everything together, it is clear that amino acids might be used as promising biomarkers in order to diagnose the specific IBD suffered by one patient and even analyse the specific clinical disease activity.

## 5. Food Components and Xenobiotics

The metabolomic analysis in IBD patients have also identified a huge diversity of xenobiotic compounds and their related metabolites, which are differentially present in these patients. The concept of xenobiotic comprises all the chemical substances found within an organism, which are foreign to the body or to an ecological system. These chemical products include plant constituents, cosmetics, drugs, food additives, industrial chemicals and environmental pollutants, and they access the organisms through several ways such as the air, diet, drinking water, lifestyle or drug administration [95]. Given the importance of the environment in both the onset and the evolution of IBD, the xenobiotics play a pivotal role in this pathology and might provide essential information regarding the molecular mechanisms involved in this disease. Nevertheless, to what extent they play beneficial or harmful effects in this pathology remains poorly characterized. Therefore, in this section, we describe the xenobiotics, which have been identified specifically in IBD patients so far, and we discuss whether they can be considered biomarkers or pharmacological targets.

The diet constitutes one of the most critical environmental factor with a huge impact on the composition of intestinal microbiota affecting the immune response [96]. In addition, given the introduction of the modern processed foods, the amount of xenobiotcs introduced in our body throughout our life is continuously increasing [97]. Indeed, it has been described that children with CD present changes in the gut microbiome and xenobiotic metabolism [98]. Given the fact that coffee, one of the most commonly consumed drinks, contains a wide range diversity of bioactive compounds such as caffeine, trigonelline, phenolic compounds, diterpenes and soluble fibre; the coffee-derived metabolites have also been analysed in IBD patients [99]. One example of a specific xenobiotic specifically found in these patients is the major caffeine metabolite called 5-acetylamino-6-amino-3-methyluracil (AAMU). In fact, Lai and colleagues have recently reported that levels of AAMU are significantly increased in the serum of active CD compared with inactive CD patients and the control group. During the metabolism of caffeine, xanthine oxidoreductase catalyses paraxanthine to xanthine, which is oxidized in uric acid [100]. Interestingly, Neubauer and colleagues have recently analysed the levels of uric acid in serum of both UC and CD patients, and they reported significantly lower levels only in both active CD and active UC patients compared with control groups. In the same study, they also reported lower levels of free thiol status and total antioxidant status in both CD and UC patients [101].

A special consideration of the vitamin D metabolites needs to be addressed, since the vitamin D deficiency is more prevalent in patients with CD or UC than in non-IBD patients [102]. There is growing evidence that reduced serum levels of vitamin D are associated with an increased disease activity in IBD patients [103,104,105]. Furthermore, Limketkai and colleagues have recently expanded these observations, and they have reported that the reduced levels of serum vitamin D are observed once the IBD disease has been diagnosed and not before the diagnosis, which suggests that reduced levels of vitamin D are not the cause of the onset of the inflammation, but the chronic inflammation leads to lower levels of vitamin D [106]. Nevertheless, although the quantification of vitamin D is based on the determination of levels of total 25-hydroxyvitamin D (25OHD), other vitamin D metabolites such as 1,25OH2D and dihydroxycholecalciferol (24,25OH2D) are also of potential interest, since 25OHD is not the most biologically active metabolite [107]. In fact, the utility of serum 25OHD levels as biomarker for IBD patients is still not clear. In this line, Aksan and colleagues have recently reported a study in which they analysed the serum levels of 25OHD, vitamin-D-binding protein (VDBP), 1,25-dihydroxyvitamin D (1,25OHD) and dihydroxycholecalciferol (24,25OHD) in a cohort of IBD patients and demonstrated that serum total 25OHD was the only vitamin D metabolite without a correlation with the levels of inflammatory parameters. Hence, in this study, authors suggest that total 25OHD seems to be the best marker of vitamin D concentration in IBD patients regardless of the inflammatory status [108]. However, there are still several questions that still need an answer: Is the supplementation of vitamin D beneficial for IBD patients? Can the reduced levels of vitamin D trigger the inflammation or are these lower levels the consequence of the chronic inflammation observed in these patients? Do vitamin D levels correlate with the clinical activity of the disease? It is clear that, in spite of the huge number of studies analysing vitamin D levels in IBD patients, additional studies must be performed with the purpose to answer all these concerns.

Besides vitamin D, accumulative evidence has demonstrated that IBD patients also present reduced levels of other vitamins such as vitamin B9 (folate) and vitamin B12 (cobalamin), whose deficiency causes a worsening in the clinical, biochemical and inflammatory status [109,110]. In line with this, Gioxari and colleagues have recently reproduced these observations in a cohort of Greek IBD patients where they reported a serum deficiency in vitamin D, B9 and B12 in 36.8, 18.4 and 5.7% of patients, respectively. They also correlated these serum metabolite levels with clinical information and pointed to the serum vitamin profile as a complementary biomarker for the evaluation of the disease activity [111]. On the other hand, Weng and colleagues have recently reported that the levels of other vitamins compounds such as vitamin B2 (riboflavin) and nicotinate are significantly reduced in faecal samples from both UC and CD patients compared with healthy controls. In the same study, authors also correlated the metabolite levels with the abundance of intestinal microbiota, and they identified eight key species that were associated with 25(OH)D3, 3-hydroxyisovaleric acid, ascorbylstearate, glycolithocholic acid, nicotinate and putrescine. Moreover, they also showed a negative correlation between Clostridium clostridioforme and vitamin D levels, while Alcanivorax hongdengensi positively correlated with vitamin C [27]. It is important to take into account that this study reveals the direct influence of the intestinal microbiota in the metabolomics profiles.

Apart from metabolites derived from coffee and several vitamins, any food can be the source of different xenobiotics. For instance, Lai and colleagues have recently reported that the levels of pterosin E from root vegetables are significantly increased in the serum of both active and inactive CD patients compared with the control group [23]. In addition, Kestheli and colleagues compared the metabolomic signatures in urine samples from postoperative recurrent CD patients and CD patients in remission, and they reported increased levels of 1,6-anhydro-beta-D-Glucose (levoglucosan), an organic compound with a six-carbon ring structure formed after the pyrolysis of carbohydrates, and reduced levels of propylene glycol in recurrent CD patients. In addition, in this study, the authors also demonstrated that levoglucosan levels correlated with the presence of Bacteroidales and gammaproteobacteria, which strongly supports the direct relationship between the intestinal microbiota and the presence of xenobiotics in urine of IBD patients [112].

Altogether, given the scarce number of studies analysing the presence of xenobiotics in IBD patients and the huge diversity of these chemical compounds, future studies are needed in order to (a) detect key xenobiotics, which might be useful to identify environmental risk factors associated to this pathology, (b) find potential biomarkers or pharmacological targets for IBD patients, (c) confirm whether these differences are specific to IBD patients or are only due to differences in the lifestyle of the patients analysed so far, (d) validate the data already published in several cohort of IBD patients, (e) assess the efficacy of the pharmacological treatment.

## 6. Conclusions

IBD is a chronic pathology whose aetiology is still not well known and the clinical use of biomarkers in order to diagnose this pathology or evaluate the disease activity is still scarce due to the lack of reliable biomarkers. Of interest, during the last years, metabolomics has bounced into IBD and has identified specific metabolomic profiles in these patients. Thanks to the identification of numerous metabolites differentially present in IBD patients, metabolomics has become an extremely useful technique in order to identify promising biomarkers that might provide better tools for both the diagnosis and the evaluation of this disease.

Interestingly, the metabolomic studies performed so far have identified a huge number of metabolites, whose levels are specifically different in IBD patients, involved in a wide range of metabolic pathways such as TCA cycle, β-oxidation, urea cycle, etc. The direct effect of intestinal inflammation on several metabolic pathways is widely assumed [113]. In this point, it is important to take into account that those metabolites are usually involved in molecular pathways implicated in the inflammatory response. Nevertheless, it is still not clear whether this metabolic alteration is a cause or a consequence of the chronic and persistent inflammation present in IBD patients. Although emerging studies suggest that these metabolic disturbances might appear as a result of an inflammatory scenario, further studies are needed in order to confirm this hypothesis.

One important aspect that needs to be considered is the type of biological sample used in the metabolomic analysis. During this review, we have detailed the kind of sample used in each study, since the levels of each metabolite can differ depending on the biological sample due to the microbial dysbiosis, impaired intestinal absorption or even pharmacological and surgical treatments of IBD patients. To date, most of the studies performed have analysed the metabolome specifically in non-invasive biological samples such as serum, plasma, urine and stool. Nevertheless, the analysis of the metabolomics profiles specifically in intestinal tissue of IBD patients would reflect the specific metabolic alterations in this tissue and might provide more specific information about possible biomarkers or even pharmacological targets. Therefore, despite the difficulty in obtaining intestinal tissues, future studies are needed in order to better elucidate the molecular mechanisms involved in the pathogenesis of IBD.

At this point, we would like to mention that one limitation of the metabolomics studies is the reduced number of IBD patients included. Hence, bigger cohort of patients must be studied in the next few years with the purpose of identifying specific metabolites that can be used in the clinics as biomarkers. In addition, one of the most pursued aims is to identify biomarkers that can predict whether an IBD patient will be a responder or a non-responder to one conventional therapy. Of interest, Ding and colleagues have recently reported a set of metabolites involving lipid, bile acid and amino acid pathways, which might contribute to predict the response to anti-TNF therapy [114]. This elegant study represents the starting point of a promising strategy in order to stablish some metabolites as predictors of the efficacy of the pharmacological treatment assigned to each IBD patient. Nevertheless, to achieve this ambitious goal, additional studies are required so as to confirm the potential capacity of those metabolites to predict the response of each IBD patient to a conventional therapy.

Finally, it is important to take into account that, despite the large number of metabolites identified so far, there is still a huge gap between the identification of these compounds and the knowledge of their specific role in IBD. Hence, future studies should integrate all the metabolomic profiles with other results derived from different omics. Thus, these studies would identify new biomarkers and molecular mechanisms involved in the pathogenesis of IBD.

## Figures and Tables

**Table 1:** Differential abundance of tricarboxylic acid cycle (TCA) cycle intermediates in IBD patients. Crohn´s disease (CD), ulcerative colitis (UC), colon carcinoma patients (CCR), ultra-high performance liquid chromatography/tandem mass spectroscopy (UPLC-MS/MS), proton nuclear magnetic resonance (^1^H-NMR), gas chromatography/mass spectrometry (GC/MS).

Metabolite	Alteration	Patients	Technique	Sample	Reference
**Citrate**	Decreased	CD vs. UC and control	UPLC-MS/MS	Serum	[10]
	Decreased	CD and UC vs. control	^1^H-NMR	Urine	[11]
	Decreased	IBD vs. non-IBD	^1^H-NMR	Serum and Urine	[12]
	Increased	Active IBD vs. IBD in remission	^1^H-NMR	Serum and Urine	[12]
	Decreased	CD and UC vs. control	^1^H-NMR	Serum and Urine	[8]
	Increased	CD vs. UC	^1^H-NMR	Serum	[8]
	Decreased	IBD vs. non-IBD	NMR	Urine	[9]
	Decreased	Damaged vs. non-damaged UC tissue	GC/MS	Intestinal Biopsies	[16]
	Unchanged	Paediatric IBD vs. paediatric non-IBD	^1^H-NMR	Faecal	[14]
**Aconitate**	Decreased	CD vs. UC and control	UPLC-MS/MS	Serum	[10]
	Decreased	IBD vs. non-IBD	NMR	Urine	[9]
**α-ketoglutarate**	Decreased	CD vs. UC and control	UPLC-MS/MS	Serum	[10]
**Succinate**	Decreased	CD vs. UC and control	UPLC-MS/MS	Serum	[10]
	Decreased	IBD vs. non-IBD Active IBD vs. IBD in remission	^1^H-NMR	Urine	[12]
	Decreased	CD and UC vs. control	^1^H-NMR	Urine	[8]
	Decreased	IBD vs. non-IBD	NMR	Urine	[9]
	Decreased	CD and UC vs. non-IBD	^1^H-NMR	Intestinal Biopsies	[15]
	Decreased	Damaged vs. non-damaged UC tissue	GC/MS	Intestinal Biopsies	[16]
	Increased	CD vs. healthy CCR patients	^1^H-NMR	Intestinal Resections	[17]
	Unchanged	Paediatric IBD vs. paediatric non-IBD	^1^H-NMR	Faecal	[14]
	Unchanged	UC and CD vs. control	^1^H-NMR	Faecal	[13]
**Fumarate**	Decreased	CD vs. UC and control	UPLC-MS/MS	Serum	[10]
	Decreased	Damaged vs. non-damaged UC tissue	GC/MS	Intestinal Biopsies	[16]
**Malate**	Decreased	CD vs. UC and control	UPLC-MS/MS	Serum	[10]
	Decreased	Damaged vs. non-damaged UC tissue	GC/MS	Intestinal Biopsies	[16]
**Isocitrate**	Decreased	Damaged vs. non-damaged UC tissue	GC/MS	Intestinal Biopsies	[16]

**Table 2:** Differential abundance of fatty acids in IBD patients. Crohn´s disease (CD), ulcerative colitis (UC), predominantly ileal CD (ICD), predominantly colonic CD (CCD), ultra-high performance liquid chromatography/tandem mass spectroscopy (UPLC-MS/MS), proton nuclear magnetic resonance (^1^H-NMR), gas chromatography/mass spectrometry (GC/MS), liquid chromatography/mass spectrometry (LC/MS), ion cyclotron resonance Fourier transform mass spectrometry (ICR-FT/MS).

	Metabolite	Alteration	Patients	Technique	Sample	Reference
**Long-Chain Fatty Acids (LCFAs)**	**Unspecified LCFAs**	Decreased	IBD vs. control	UPLC-MS/MS	Serum	[10]
		Decreased	CD vs. UC	UPLC-MS/MS	Serum	[10]
	**Docosahexaenoic Acid**	Decreased	Active CD and Inactive CD vs. control	LC/MS	Serum	[23]
		Decreased	Paediatric UC vs. control	LC/MS	Serum	[24]
	**Linolenic Acid**	Decreased	Active CD and Inactive CD vs. control	LC/MS	Serum	[23]
		Increased	ICD vs. CCD and control	ICR-FT/MS	Faeces	[26]
	**Arachidonic Acid**	Decreased	Active CD and Inactive CD vs. control	LC/MS	Serum	[23]
		Decreased	Paediatric UC vs. control	LC/MS	Serum	[24]
		Increased	ICD vs. CCD and control	ICR-FT/MS	Faeces	[26]
	**Arachidic Acid**	Decreased	IBD vs. control	GC/MS	Faeces	[27]
	**Oleic Acid**	Decreased	IBD vs. control	GC/MS	Faeces	[27]
		Increased	ICD vs. CCD and control	ICR-FT/MS	Faeces	[26]
	**Tridecanoic Acid**	Decreased	IBD vs. control	GC/MS	Faeces	[27]
	**Palmitic Acid**	Increased	ICD vs. CCD and control	ICR-FT/MS	Faeces	[26]
	**Stearic Acid**	Increased	ICD vs. CCD and control	ICR-FT/MS	Faeces	[26]
	**6Z-, 9Z-, 12Z Octadecatrienoic Acid**	Increased	ICD vs. CCD and control	ICR-FT/MS	Faeces	[26]
	**2-Hydroxymyristic acid**	Decreased	IBD vs. control	LC/MS	Faeces	[33]
**Medium-Chain Fatty Acids**	**Pelargonic Acid**	Decreased	Active CD and Inactive CD vs. control	LC/MS	Serum	[23]
	**Caprylic Acid**	Decreased	Active CD and Inactive CD vs. control	LC/MS	Serum	[23]
		Decreased	IBD vs. control	LC/MS	Faeces	[33]
	**Sebacic Acid**	Decreased	IBD vs. control	GC/MS	Faeces	[27]
	**Isocaproic Acid**	Decreased	IBD vs. control	GC/MS	Faeces	[27]
**Short-Chain Fatty Acids**	**Butyrate**	Decreased	IBD vs. control	^1^H-NMR	Faeces	[13]
		Decreased	IBD vs. control	LC/MS	Faeces	[33]
		Decreased	Active CD vs. control	^1^H-NMR	Faeces	[39]
	**Propionate**	Decreased	IBD vs. control	LC/MS	Faeces	[33]
		Decreased	Active CD vs. control	^1^H-NMR	Faeces	[39]
	**Acetate**	Decreased	IBD vs. control	^1^H-NMR	Urine	[9]
		Decreased	IBD vs. control	^1^H-NMR	Faeces	[13]
**Polyunsaturated Fatty Acids**	**Unspecified Polyunsaturated Fatty Acids**	Decreased	IBD vs. control CD vs. UC	UPLC-MS/MS	Serum	[10]
		Decreased	IBD vs. control CD vs. UC	^1^H-NMR	Serum	[42]
	**Eicosatrienoic**	Increased	IBD vs. control	LC/MS	Faeces	[33]
	**Docosapentaenoic**	Increased	IBD vs. control	LC/MS	Faeces	[33]
**Branched-Chain Fatty acids**		Decreased	IBD vs. control CD vs. UC	UPLC-MS/MS	Serum	[10]

**Table 3:** Differential abundance of sterol lipids in IBD patients. Crohn´s disease (CD), ulcerative colitis (UC), predominantly ileal CD (ICD), predominantly colonic CD (CCD), ultra-high performance liquid chromatography/tandem mass spectroscopy (UPLC-MS/MS), proton nuclear magnetic resonance (^1^H-NMR), gas chromatography/mass spectrometry (GC/MS), liquid chromatography/mass spectrometry (LC/MS), ion cyclotron resonance Fourier transform mass spectrometry (ICR-FT/MS).

	Metabolite	Alteration	Patients	Technique	Sample	Reference
**Bile Acids**	**Unespecified Bile Acids**	Increased	CD vs. control	UPLC-MS/MS	Serum	[10]
		Decreased	UC vs. control	UPLC-MS/MS	Serum	[10]
	**Glycochenodeoxycholate glucuronide**	Decreased	UC vs. control	UPLC-MS/MS	Serum	[10]
	**Deoxycholate**	Decreased	UC vs. control	UPLC-MS/MS	Serum	[10]
	**Taurolithocholate 3-sulphate**	Decreased	UC vs. control	UPLC-MS/MS	Serum	[10]
	**Cholate**	Increased	CD vs. control	LC/MS	Faeces	[33]
	**Chenodeoxycholate**	Increased	CD vs. control	LC/MS	Faeces	[33]
	**Glycocholate**	Increased	CD vs. control	ICR-FT/MS	Faecal	[26]
	**Taurocholate**	Increased	ICD vs. CCD and control	ICR-FT/MS	Faecal	[26]
	**3** **α** **, 7** **α** **, 12** **α** **-trihydroxy-5** **β** **-cholanate**	Increased	ICD vs. CCD and control	ICR-FT/MS	Faecal	[26]
	**Chenodeoxyglycocholate**	Increased	ICD vs. CCD and control	ICR-FT/MS	Faecal	[26]
	**Glycochenoeoxycholate glucuronide**	Decreased	UC vs. control	UPLC-MS/MS	Serum	[10]
	**Glycochenoeoxycholate**	Decreased	UC vs. control	GC/MS	Faeces	[27]
	**Glycolithocholate**	Increased	CD vs. control	UPLC-MS/MS	Serum	[10]
		Decreased	UC vs. control	GC/MS	Faeces	[27]
	**Glycoursodeoxycholate**	Increased	CD vs. control	UPLC-MS/MS	Serum	[10]
	**Ursodeoxycholate**	Increased	CD vs. control	UPLC-MS/MS	Serum	[10]
	**Lithocholic acid**	Decreased	IBD vs. control	GC/MS	Faeces	[27]
		Decreased	CD vs. control	LC/MS	Faeces	[33]
	**Chenodeoxycholate**	Decreased	IBD vs. control	GC/MS	Faeces	[27]
	**Taurolithocholic acid**	Decreased	IBD vs. control	GC/MS	Faeces	[27]
		Decreased	CD vs. control	UPLC-MS/MS	Serum	[10]
	**Taurocholenate sulphate**	Decreased	CD vs. control	UPLC-MS/MS	Serum	[10]
	**Deoxycholate**	Decreased	UC vs. control	UPLC-MS/MS	Serum	[10]
		Decreased	CD vs. control	LC/MS	Faeces	[33]
**Cholesterol**	**Unspecified Cholesterol**	Decreased	CD vs. control	LC/MS	Faeces	[33]
	**LDL cholesterol**	Decreased	IBD vs. control	^1^H-NMR	Serum	[42]
			CD vs. UC			
		Decreased	IBD active vs. IBD remission and control	^1^H-NMR	Serum	[12]
	**HDL cholesterol**	Decreased	IBD vs. control	^1^H-NMR	Serum	[42]
			CD vs. UC			
	**VLDL cholesterol**	Increased	IBD vs. control	^1^H-NMR	Serum	[42]
			CD vs. UC			
		Decreased	IBD active vs. IBD remission and control	^1^H-NMR	Serum	[12]

**Table 4:** Differential abundance of sphingolipids, glycerophospholipids, glycerolipids, prenol lipids and choline in IBD patients. Crohn´s disease (CD), ulcerative colitis (UC), ultra-high performance liquid chromatography/tandem mass spectroscopy (UPLC-MS/MS), proton nuclear magnetic resonance (^1^H-NMR), liquid chromatography/mass spectrometry (LC/MS).

	Metabolite	Alteration	Patients	Technique	Sample	Reference
**Sphingolipids**	**Ceramide**	Increased	CD vs. control	LC/MS	Faeces	[33]
	**Lactosylceramide**	Increased	Paediatric CD vs. paediatric UC	LC/MS	Serum	[24]
			Paediatric CD vs. paediatric UC Paediatric IBD vs. control	UPLC-MS/MS	Serum	[65]
	**Sphingomyelin**	Increased	CD vs. control	LC/MS	Faeces	[33]
		Decreased	Paediatric CD vs. control	LC/MS	Serum	[24]
		Decreased	UC vs. control	UPLC-MS/MS	Serum	[10]
	**Sphingosine-1-phosphate**	Decreased	Active CD and inactive CD vs. control	LC/MS	Serum	[23]
**Glycerophospholipids**	**Lysophosphatidylcholine** **Phosphatidylcholine,** **Phosphatidylethanolamine, Lysophosphatidylethanolamine**	Decreased	Paediatric IBD vs. control	LC/MS	Serum	[24]
	**Lysophosphatidylcholine**	Decreased	Paediatric UC vs. control	LC/MS	Serum	[24]
	**Glycerophosphorylcholine**	Decreased	IBD vs. control Remission IBD vs. control Active CD vs. remission CD	^1^H-NMR	Intestinal Biopsies	[15]
**Glycerolipids**	**Glycerol**	Decreased	IBD vs. control	UPLC-MS/MS	Serum	[10]
		Increased	CD vs. UC and control	^1^H-NMR	Faeces	[13]
	**Triacylglycerols**	Decreased	IBD vs. control	LC/MS	Faeces	[33]
**Prenol lipids**	**Triterpenoids**	Decreased	IBD vs. control	LC/MS	Faeces	[33]
**Choline**		Decreased	CD vs. UC	^1^H-NMR	Serum	[42]
		Decreased	IBD vs. control	^1^H-NMR	Serum	[42]
		Decreased	IBD vs. control	^1^H-NMR	Serum and plasma	[8]
		Decreased	IBD vs. control	^1^H-NMR	Serum	[12]
		Decreased	IBD vs. control	^1^H-NMR	Colonic mucosa	[15]
		Decreased	Active CD vs. remission CD	^1^H-NMR	Colonic mucosa	[15]

**Table 5:** Differential abundance of amino acids in IBD patients. Crohn´s disease (CD), ulcerative colitis (UC), predominantly ileal CD (ICD), ultra-high performance liquid chromatography/tandem mass spectroscopy (UPLC-MS/MS), proton nuclear magnetic resonance (^1^H-NMR), liquid chromatography/mass spectrometry (LC/MS), ion cyclotron resonance Fourier transform mass spectrometry (ICR-FT/MS).

Metabolite	Alteration	Patients	Technique	Sample	Reference
**5-Hydroxy-L-tryptophan**	Decreased	Active CD and inactive CD vs. control	LC/MS	Serum	[23]
**Tryptophan**	Increased	UC vs. control	^1^H-NMR	Urine	[8]
	Increased	ICD vs. control	ICR-FT/MS	Faecal	[26]
**Indole-3-propionic acid**	Decreased	Active CD and inactive CD vs. control	LC/MS	Serum	[23]
**Indole-3-acrylic acid**	Decreased	Active CD and inactive CD vs. control	LC/MS	Serum	[23]
**3-Methylindole**	Decreased	Active CD and inactive CD vs. control	LC/MS	Serum	[23]
**Phenylalanine**	Increased	ICD vs. control	ICR-FT/MS	Faeces	[26]
	Increased	IBD vs. control	^1^H-NMR	Faeces	[39]
	Decreased	Active CD and inactive CD vs. control	LC/MS	Serum	[23]
	Increased	IBD vs. control Active IBD vs. remission IBD	^1^H-NMR	Serum	[12]
**Ergothioneine**	Decreased	Active CD and inactive CD vs. control	LC/MS	Serum	[23]
**Histidine**	Decreased	IBD vs. control	^1^H-NMR	Urine	[9]
	Decreased	IBD vs. control	^1^H-NMR	Serum	[12]
**Tyrosine**	Decreased	IBD vs. control	^1^H-NMR	Serum and Plasma	[8]
	Increased	IBD vs. control	^1^H-NMR	Faeces	[13]
	Increased	IBD vs. control	^1^H-NMR	Faeces	[39]
**Dopaquinone**	Increased	CD vs. control	ICR-FT/MS	Faeces	[26]
**Leucine**	Decreased	CD vs. control CD vs. UC	UPLC-MS/MS	Serum	[10]
	Increased	IBD vs. control Active UC vs. inactive UC and control	^1^H-NMR	Faeces	[39]
	Increased	IBD vs. control	^1^H-NMR	Serum	[12]
	Increased	IBD vs. control	^1^H-NMR	Faeces	[13]
	Decreased	IBD vs. control Active CD vs. remission CD	^1^H-NMR	Intestinal Biopsies	[15]
**Isoleucine**	Decreased	IBD vs. control	^1^H-NMR	Serum	[42]
	Increased	IBD vs. control Active UC vs. inactive UC and control	^1^H-NMR	Faeces	[39]
	Increased	IBD vs. control	^1^H-NMR	Serum and Plasma	[8]
	Increased	IBD vs. control	^1^H-NMR	Serum	[12]
	Increased	IBD vs. control	^1^H-NMR	Faeces	[13]
	Decreased	IBD vs. control Active CD vs. remission CD	^1^H-NMR	Intestinal Biopsies	[15]
**Valine**	Decreased	CD vs. control CD vs. UC	UPLC-MS/MS	Serum	[10]
	Increased	IBD vs. control Active UC vs. inactive UC and control	^1^H-NMR	Faeces	[39]
	Decreased	IBD vs. control	^1^H-NMR	Serum and Plasma	[8]
	Increased	IBD vs. control	^1^H-NMR	Faeces	[13]
	Decreased	IBD vs. control Active CD vs. remission CD	^1^H-NMR	Intestinal Biopsies	[15]
**Glutamine**	Decreased	CD vs. control CD vs. UC	UPLC-MS/MS	Serum	[10]
	Decreased	IBD vs. control	^1^H-NMR	Intestinal Biopsies	[15]
**Alanine**	Decreased	IBD vs. control	^1^H-NMR	Serum	[42]
	Increased	IBD vs. control	^1^H-NMR	Faeces	[13]
	Increased	CD vs. UC	^1^H-NMR	Serum	[12]
	Increased	Active IBD vs. remission IBD	^1^H-NMR	Urine	[12]
	Increased	IBD vs. control Active UC vs. inactive UC and control	^1^H-NMR	Faeces	[39]
	Decreased	IBD vs. control	^1^H-NMR	Intestinal Biopsies	[15]
**Lysine**	Decreased	CD vs. control	UPLC-MS/MS	Serum	[10]
	Increased	IBD vs. control	^1^H-NMR	Serum and Plasma	[8]
	Decreased	IBD vs. control	^1^H-NMR	Urine	[9]
	Increased	IBD vs. control	^1^H-NMR	Faeces	[13]
	Increased	IBD vs. control	^1^H-NMR	Faeces	[39]
		Active UC vs. inactive UC and control			
**Glycylproline**	Increased	UC vs. control	^1^H-NMR	Urine	[8]
**Arginine**	Decreased	CD vs. control	UPLC-MS/MS	Serum	[10]
	Increased	IBD vs. control	^1^H-NMR	Serum and Plasma	[8]
**Ornithine**	Decreased	CD vs. control	^1^H-NMR	Serum and Plasma	[8]
**Methionine**	Increased	IBD vs. control	^1^H-NMR	Serum and Plasma	[8]
**Serine**	Decreased	CD vs. control CD vs. UC	UPLC-MS/MS	Serum	[10]
	Decreased	IBD vs. control	^1^H-NMR	Serum and Plasma	[8]
**Glycine**	Increased	IBD vs. control	^1^H-NMR	Serum and Plasma	[8]
	Increased	IBD vs. control	^1^H-NMR	Serum	[12]
	Increased	Active IBD vs. remission IBD	^1^H-NMR	Urine	[12]
	Decreased	Remission IBD vs. control	^1^H-NMR	Urine	[12]
	Increased	IBD vs. control	^1^H-NMR	Faeces	[39]
**Asparagine**	Decreased	IBD vs. control	^1^H-NMR	Urine	[9]
	Increased	IBD vs. control	^1^H-NMR	Faeces	[13]
**Aspartic acid**	Increased	IBD vs. control	^1^H-NMR	Faeces	[13]
**Glutamate**	Increased	IBD vs. control	^1^H-NMR	Faeces	[13]
	Decreased	IBD vs. control	^1^H-NMR	Intestinal Biopsies	[15]
**Proline**	Increased	IBD vs. control	^1^H-NMR	Serum and Plasma	[8]
**Taurine**	Decreased	IBD vs. control Remission IBD vs. control	^1^H-NMR	Urine	[12]
	Increased	Active UC vs. inactive UC and control	^1^H-NMR	Faeces	[39]

## Data Availability

Not applicable.
